# Omega-3 fatty acids are protective against paclitaxel-induced peripheral neuropathy: A randomized double-blind placebo controlled trial

**DOI:** 10.1186/1471-2407-12-355

**Published:** 2012-08-15

**Authors:** Zohreh Ghoreishi, Ali Esfahani, Abolghasem Djazayeri, Mahmoud Djalali, Banafsheh Golestan, Hormoz Ayromlou, Shahriar Hashemzade, Mohammad Asghari Jafarabadi, Vahid Montazeri, Seyed Ali Keshavarz, Masoud Darabi

**Affiliations:** 1 Department of Nutrition and Biochemistry, School of Health, Tehran University of Medical Sciences, Tehran, Iran; 2 Hematology and Oncology Research Center, Tabriz University of Medical Sciences, Tabriz, Iran; 3 Department of Epidemiology and Biostatistics, Tehran University of Medical Sciences, Tehran, Iran; 4 Neurology Research Center, Tabriz University of Medical Sciences, Tabriz, Iran; 5 Department of General Surgery, School of Medicine, Tabriz University of Medical Sciences, Tabriz, Iran; 6 Tabriz Health Services Management research Center and Department of Statistics and Epidemiology, Faculty of Health and Nutrition, Tabriz University of Medical sciences, Tabriz, Iran; 7 Department of Biochemistry, School of Medicine, Tabriz University of Medical sciences, Tabriz, Iran

**Keywords:** Breast cancer, Omega-3 fatty acids, Paclitaxel, Peripheral neuropathy

## Abstract

**Background:**

Axonal sensory peripheral neuropathy is the major dose-limiting side effect of paclitaxel.Omega-3 fatty acids have beneficial effects on neurological disorders from their effects on neurons cells and inhibition of the formation of proinflammatory cytokines involved in peripheral neuropathy.

**Methods:**

This study was a randomized double blind placebo controlled trial to investigate the efficacy of omega-3 fatty acids in reducing incidence and severity of paclitaxel-induced peripheral neuropathy (PIPN). Eligible patients with breast cancer randomly assigned to take omega-3 fatty acid pearls, 640 mg t.i.d during chemotherapy with paclitaxel and one month after the end of the treatment or placebo. Clinical and electrophysiological studies were performed before the onset of chemotherapy and one month after cessation of therapy to evaluate PIPN based on "reduced Total Neuropathy Score".

**Results:**

Twenty one patients (70%) of the group taking omega-3 fatty acid supplement (n = 30) did not develop PN while it was 40.7%( 11 patients) in the placebo group(n = 27). A significant difference was seen in PN incidence (OR = 0.3, .95% CI = (0.10-0.88), p = 0.029). There was a non-significant trend for differences of PIPN severity between the two study groups but the frequencies of PN in all scoring categories were higher in the placebo group (0.95% CI = (−2.06 -0.02), p = 0.054).

**Conclusions:**

Omega-3 fatty acids may be an efficient neuroprotective agent for prophylaxis against PIPN. Patients with breast cancer have a longer disease free survival rate with the aid of therapeutical agents. Finding a way to solve the disabling effects of PIPN would significantly improve the patients’ quality of life.

**Trial registration:**

This trial was registered at ClinicalTrials.gov (NCT01049295)

## Background

Paclitaxel is one of the taxane-derived chemotherapeutic agents used for treatment of solid tumors including those of the breast, ovary, lung, and Kaposi’s sarcoma. It was originated from the Pacific yew *Taxus brevifolia*. Paclitaxel is an antimicrotubular agent that polymerizes tubulin, resulting in formation of stabilized disordered microtubules without dynamic instability that holds up cell division [1,2].

Paclitaxel-induced peripheral neuropathy (PIPN) is the major dose-limiting side effect of paclitaxel [2]. The precise pathology of PIPN is not fully understood. Paclitaxel induces an axonal sensory peripheral neuropathy (PN) as a result of aggregation of microtubules in axons and Schwann cells. It may also cause fiber demyelination in some severs cases [1].

Signs of paclitaxel neurotoxicity usually take three weeks to appear, mainly affecting the sensory, rather than motor or autonomic nervous systems. The most common symptoms of PIPN include numbness, tingling, paresthesias, and a burning pain in a stocking-glove distribution. The onset of symptoms is often in hands and feet simultaneously, and some patients complain of facial discomfort [3,4].

Omega-3 fatty acids eicosapentaenoic acid ( EPA) and docosahexaenoic acid (DHA) are polyunsaturated fatty acids(PUFAs) incorporated into the phospholipid membrane of cells including those of the central and peripheral nervous systems [5]. They have many beneficial effects in some psychiatric and neurodegenerative diseases. They determine the biophysical properties of neuronal membranes and regulate signal transduction by their effect on ion channels and receptor functions [6]. Moreover, the production of proinflammatory cytokines which induce neuropathy is attenuated by EPA and DHA, in particular DHA [5,6]. DHA-induced myelinogenesis has been reported in the previous studies [7].

Most adverse effects associated with chemotherapy are ameliorated after cessation of the therapy, but PN maybe partly reversible or even irreversible in some patients and as such can significantly decrease the patients’ quality of life. Taken together, we designed a randomized placebo-controlled trial to evaluate the possible effect of omega-3 fatty acid supplementation on PIPN in patients with breast cancer. To our knowledge, there have been no previous studies done to demonstrate the possible effects of omega-3 fatty acid supplementation on PIPN.

## Methods/design

### Trial design

**Figure 1:**
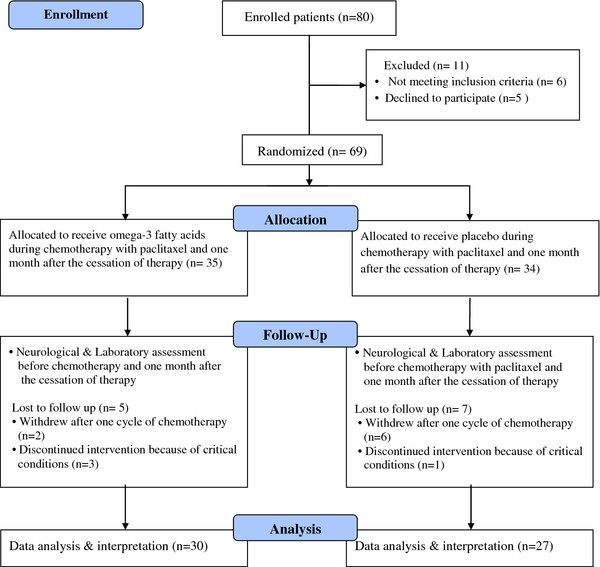
Flow diagram of the study design.

This study was a randomized double blind placebo-controlled trial. Subjects were recruited for the study from April, 2010 to October 2011 from Sheiqorrais University Clinic. The study protocol was approved by the ethical committee of Tehran University of Medical Sciences (No: 9683). Figure 1 shows a summary of the study design.

### Patient selection

Eighty patients were enrolled in the study .Eleven patients were excluded before randomization, 6 patients did not meet the inclusion criteria and 5 patients declined to participate. Therefore, 69 patients were randomly allocated to receive omega-3 fatty acids (n = 35) or placebo (n = 34). Four patients discontinued the study due to a critical health conditions and 8 patients were unwilling to continue after the first cycle of therapy (lost to follow up, n = 12). So 57 female patients completed the study (Figure 1). Inclusion criteria were: being female, age between 30 to 70 years, treatment with 4 courses of 175 mg/m^2^ paclitaxel for a positive node breast cancer, satisfactory kidney and liver function, and WHO performance scores of 0 to 1. All factors which predisposed patients to PN including a medical history of prior chemotherapy treatment, pre-existing peripheral neuropathy due to diabetes mellitus, HIV, alcohol abuse, thyroid dysfunction and hereditary PN associated disorders, and taking any form of nutritional supplement (fish oil, vitamins and minerals) at least three months before enrollment were exclusion criteria. Written informed consent was obtained from all the patients before any intervention.

### Randomization and treatment

Eligible enrolled patients were randomly assigned to receive omega-3 fatty acid oral supplements as soft gelatin capsules (Mor DHA Mini I.Q. Minami Nutrition NV, Drie Eikenstraat 661,2650 Edegem, Belgium) at a dose of 640 mg (54% DHA, 10% EPA) three times a day during chemotherapy with paclitaxel and one month after the end of therapy or placebo of Sun flower soft gelatin capsules, (Dana Pharma, Tabriz, Iran) that were similar in appearance to omega-3 fatty acid soft gelatin capsules and similarly administered. All patients treated with paclitaxel (Ebetaxel®,Ebewe Pharmaceutical Company, Austria), at a dose of 175 mg/m² given over a 3-hour infusion every three weeks for 4 cycles.

The randomization plan was based on a permuted block randomization. Sealed closed envelopes containing random codes (A or B) were used to assign subjects to either the intervention group or the control group. The allocation of patients was only known to the randomization coordinator of the study.

### Follow up

#### Primary outcome measures

All the study patients were evaluated both pre-chemotherapy and one month after the end of chemotherapy by the same neurologist who carried out both clinical examination and electrophysiologic studies and the patients’allocation to study groups was concealed to him. Reduced Total Neuropathy Score (rTNS) was used to evaluate the existence and severity of PIPN in patients as the primary outcome measures. rTNS consists of subjective sensory symptoms, pin sensibility, deep tendon reflexes, and nerve conduction studies of sural and peroneal nerves, ranging from 0–28 points. Any changes in parameters under evaluation in the tests were given a score from 0 to 4 (depending on the measured severity). By adding these seven scores together an “rTNS” score from 0 to 28 was obtained for each patient. The severity of PIPN then was graded as follows: mild (total score 1–10), moderate (total score 11-19); and severe (total score 20–28). rTNS can be easily applied in clinical settings [8]. rTNS included all the TNS items except those for motor symptoms, autonomic symptoms, and quantitative sensory testing of the vibration threshold [8]. These three items were excluded based on the following: 1) probability of occurrences with paclitaxel chemotherapy [3,4,9], and 2) the availability of the necessary device for the examination.

### Secondary outcome measures

#### Nerve conduction study

Nerve conduction studies were conducted unilaterally (right side), using a Nicolet/ VIASYS Viking Quest EMG Machine based on standard methods [10]. Distal motor latency (DML), peak to baseline amplitude of compound muscle action potential (a-CMAP), and motor conduction velocity were measured for tibial, peroneal, and ulnar nerves as the motor conduction assessment. Sensory nerve conduction of sural and ulnar nerves was evaluated with peak-to-peak amplitude measurement of sensory action potentials (a-SAP) and sensory conduction velocity (antidromic technique).

#### Serum levels of omega-3 fatty acids

Concentrations of EPA and DHA in serum phospholipids were measured by gas liquid chromatography using the method described by Noori M and his colleagues [11].

#### Statistical analysis

Data were summarized using mean (SD) and frequency (percent) for quantitative and qualitative variables respectively. Analysis of covariance was used to determine the differences of nerve conduction measurements and the serum phospholipids concentrations of omega-3 fatty acids between the two study groups, adjusting baseline measurements. To investigate the difference of PN incidence between the omega-3 supplemented group and the control group, logistic regression performed to estimate the odds ratio. Ordinal regression analysis was used to compare the severity of PN between the two study groups. All tests were two-sided and significance level was set at 0.05. Statistical power of the study was considered as 80% [12]. Analysis was done using spss software, (spss Inc., Chicago, IL).

#### Sample size

Sample size was determined based on primary information obtained from the study by Okuda et. al [13] for vibratory perception threshold(VPT): By considering α = 0.05 and a power of 80%, the sample size was computed as 23.24 (≈24) per group [12]. This number was increased to 28 per group to accommodate the anticipated 20% dropout rate, although more patients were enrolled.

## Results

Fifty seven female breast cancer patients were randomly assigned to either the omega-3 supplemented group (n = 30) or the placebo group (n = 27). From now on we pointed out the omega-3 supplemented group as group I and the placebo group as group II. The mean age of group I was 46.19 ± 9.76 and it was 45.70 ± 12.00 for group II. There was no statistical significant difference between the two groups according to age (P = 0.86). The means of body mass index (BMI) for the omega-3 supplemented group and the control group were 45.99 ± 9.01 and 44.14 ± 8.89 respectively, without statistical differences (p = 0.43). All the patients treated with 4 courses of 175 mg/m^2^ paclitaxel for a positive node breast cancer. They all had a satisfactory kidney and liver function, and WHO performance scores of 0 to 1.

### Peripheral neuropathy

The “reduced Total Neuropathy Score (rTNS)” of the 30 patients in group I was as follows: 21 patients (70%) did not develop PN and 9 patients (30%) manifested some grade of neurotoxicity: Four patients (13.3%) developed mild PN; five patients (16.7%) developed moderate PN and sever PN was not observed in this group of study subjects (Table 1).

**Table 1:** Paclitaxel-induced peripheral neuropathy in the study groups

	**Peripheral neuropathy**				
	**Normal**	**Mild**	**Moderate**	**Severe**	**Total**
Omega- 3 supplemented group	21(70%)	4(13.3%)	5(16.7%)	0%	30(100%)
Placebo received group	11(40.7%)	10(37%)	5(18.5%)	1(3.7%)	27(100%)

A significant difference in PN incidence (OR = 0.3, .95% CI = (0.10 - 0.88), p = 0.029).

No significant difference in severity of PN (B = −1.02, .95% CI = (−2.06 - 0.02), p = 0.054).

In group II, PN was not revealed in 11 patients (40.7%), while 10 patients (37%) developed mild PN and 5 patients (18.5%) developed moderate PN. Also, severe neuropathy was seen in one patient (3.7%) (Table 1). A significant difference was observed between the omega-3 supplemented group and the placebo group in PN incidence (OR = 0.3, .95% CI = (0.10-0.88), p = 0.029). So group I had 70% lower risk of PN incidence. The number needed to treat (NNT) to prevent one PN event was 3. In addition, there were not statistically significant differences in severity of PN between these two groups (B = −1.02, .95% CI = (−2.06 - 0.02), p = 0.054).

### Nerve conduction study parameters

Using analysis of covariance adjusting baseline measurements to determine the differences of quantitative values between two study groups, a significant difference of sural a-SAP was observed between the omega-3 supplemented group and the control (p = 0.015) with a sharp decrease of sural a-SAP in the placebo group. The other differences of NCS parameters did not reach statistical significance (Table 2 and 3). 

**Table 2:** Motor nerve conduction measurements

	**group**	**Pre-chemotherapy**	Post-chemotherapy^*^	***p-value***^***#***^
*Tibial nerve*				
DML(ms)	Omega 3	16.53 (1.95)	17.59 (2.21)	.870
	Placebo	17.00 (2.16)	17.80 (3.10)	
a-CMAP(mV)	Omega 3	10.00 (5.18)	11.18 (6.35)	.149
	Placebo	11.38 (6.08)	10.68 (5.80)	
MCV(m/s)	Omega 3	46.38 (3.67)	45.16 (4.24)	.359
	Placebo	46.24 (5.08)	46.03 (6.65)	
*Peroneal nerve*				
DML(ms)	Omega 3	14.16 (1.80)	14.97 (2.53)	.209
	Placebo	14.85 (3.18)	14.91 (2.37)	
a-CMAP(mV)	Omega 3	6.20 (6.68)	4.43 (3.26)	.549
	Placebo	5.51 (3.41)	3.83 (3.15)	
MCV(m/s)	Omega 3	46.13 (3.32)	45.87 (4.75)	.106
	Placebo	46.46 (5.24)	42.38 (9.90)	
*Ulnar nerve*				
DML(ms)	Omega 3	9.48 (.74)	9.67 (.86)	.439
	Placebo	9.67 (.98)	9.60 (1.29)	
a-CMAP(mV)	Omega 3	17.29 (12.95)	14.25 (3.79)	.256
	Placebo	13.80 (3.53)	13.29 (4.73)	
MCV(m/s)	Omega 3	57.30 (5.18)	55.82 (6.35)	.706
	Placebo	55.80 (4.90)	53.19 (7.29)	

DML, distal motor latency; a-CMAP, amplitude of compound muscle action potential; MCV, motor conduction velocity.

* One month after the cessation of chemotherapy.

# *p* value is reported based on the analysis of covariance.

**Table 3:** Sensory nerve conduction measurements

	**Group**	**Pre-chemotherapy**	Post-chemotherapy^*^	***p-value***^***#***^
*Sural nerve*				
a-SAP(μV)	Omega 3	13.27 (5.02)	13.33 (5.91)	.015
	Placebo	13.70 (7.46)	9.74 (5.96)	
SCV(m/s)	Omega 3	54.35 (7.13)	54.79 (8.35)	.514
	Placebo	53.50 (6.79)	52.52 (8.05)	
*Ulnar nerve*				
a-SAP(μV)	Omega 3	34.23 (14.85)	24.53 (13.95)	.454
	Placebo	31.15 (14.02)	21.53 (13.70)	
SCV(m/s)	Omega 3	57.30 (5.18)	55.82 (6.35)	.212
	Placebo	55.80 (4.90)	53.19 (7.29)	

a-SAP, sensory action potential amplitude; SCV, sensory conduction velocity.

* One month after the cessation of chemotherapy.

# *p* value is reported based on the analysis of covariance.

### Serum levels of omega-3 fatty acids

Serum phospholipid concentrations of EPA and DHA were significantly different between the omega-3 supplemented group and the placebo group (P = 0.005 and P = 0.001 respectively): in the omega-3 supplemented group, EPA concentrations increased from 0.53 ± 0.32 at the baseline to 0.61 ± 0.37 at the end of the intervention and DHA concentrations changed from 0.64 ± 48 at the baseline to 1.05 ± 0.79 after the cessation of supplementation with omega-3 fatty acids. In the placebo group, measured changes were 0.63 ± 0.39 to 0.59 ± 0.41 for EPA and 0.71 ± 0.54 to 0.63 ± 0.33 for DHA serum levels, respectively. No statistically significant differences were observed between the two study groups in incidence and severity of the PIPN according to the changes of serum DHA and EPA levels.

## Discussion

Cumulative neurotoxicity along with axonal disorders are common in PIPN [3]. The cellular microtubules of malignant cells and axons of peripheral nerves are the targets of paclitaxel and it inhibits tubulin depolymerization [4]. It was estimated that 60%-70% of patients who received this chemotherapeutic agent developed dose-dependent neurotoxicity [4,14].

The aim of the current double-blind placebo-controlled trial was to trace the efficacy of omega-3 fatty acids (mainly DHA), in prophylaxis against paclitaxel induced neurotoxicity. To do this, eligible patients with node positive breast cancer undergoing chemotherapy with paclitaxel were randomly assigned to take oral supplements of omega-3 fatty acids or a placebo during the course of their therapy cycles and one month after the end of chemotherapy.

There was a significant difference in PIPN incidence between the two study groups so that 70% of patients taking omega-3 fatty acid supplements did not develop PN while incidence was 40.7% in the placebo group. It seems that omega-3 fatty acids, in particular DHA, had neuroprotective effects and that they decreased the paclitaxel-associated neurotoxicity considerably. Our results are in accordance with previous studies that have investigated the efficacy of these fatty acids in diabetic neuropathy. They showed that omega-3 fatty acids could attenuate the severity of neuropathy in patients with type 2 diabetes mellitus [13,15]. They also prevented the lowering of nerve conduction velocity in the sciatic nerve of diabetic rats by improving the activity of Na+/K + ATPase [16]. There was a considerable trend that did not reach significance for the differences of PIPN severity between group I and II, while the frequencies of PN were higher in the placebo group almost in all scoring categories (Table 1) and severe neuropathy was not seen in the omega-3 supplemented group.

Neurophysiologic studies improve the accuracy and precision of peripheral neuropathy evaluation and help to identify patients at risk of peripheral neuropathy even before the onset of clinical symptoms [4,17]. rTNS is a composite scale used to assess the incidence and severity of PIPN that can be easily used in the research and clinical centers [8]. Generally, rTNS is well correlated with the oncologic toxicity scales including National Cancer Institute- Common Toxicity Criteria (NCI-CTS), Eastern Cooperative Oncology Group (ECOG), Ajani, and the extended TNS version with these additional parameters: motor symptoms, autonomic symptoms and quantitative sensory testing (QST) [8].

PIPN is associated with a decrease of a-SAP without significant changes of nerve conduction velocity as it was seen in this study and these changes are indicators of axonal dysfunction rather than myelin disorders [4]. In the current study, a considerable difference was observed in sural nerve a-SAP between the two groups with a sharp decrease in the placebo group. Argyriou et al. [18], evaluated the role of clinical and NCS measurements to predict the outcomes of CIPN. They found that only the decrease of the sural a-SAP was associated with the worse neurological outcomes. Our results showed that omega-3 fatty acids prevented the significant decrease of sural nerve a-SAP in group I that it may be related to their ability of PIPN reduction in this group.

Previous studies have shown that there are no known pharmacologic agents to prevent or to cure PN in cancer patients. In recent years, the efficacy of some vitamins and minerals, amino acids, cytokines, carnitines, antidepressants and anticonvulsants have been tested [19]. Vitamin E, acetyl-L carnitine, and glutamine are among the oral supplements which have been studied to prevent or attenuate PIPN, but they were not evaluated in large randomized placebo-controlled trials or they had little success in this regard [19]. An ideal neuroprotective agent for prophylaxis against PIPC should be safe for the patients without reducing the efficacy of the therapy, DHA may be the answer.

Growing evidences have demonstrated the positive influences of omega-3 fatty acids in the prevention of a wide range of psychiatric, arrhythmic, and neurological disorders such as Alzheimer’s and Parkinson’s diseases, major depression. schizophrenia and dementia. Omega-3 fatty acids are a branch of long chain polyunsaturated fatty acids, originated from marine and plant sources. By incorporating into the neuronal cell membrane phospholipids, they influence critical membrane-associated functions like signal transduction, ion channel dependent transportations (via voltage-dependent sodium channels and L-type calcium channels), receptors physiological properties, and neurotransmission [6,20]. In addition to their direct effect on neuropathic pain, omega-3 fatty acids inhibit the production of proinflammatory cytokines associated with neuropathic pain (i.e., IL-1β, IL-6, and TNF-α) [5], and the role of DHA in myelogenesis has been documented in the previous studies [6,7,20]. Lauretania F et.al, have shown that DHA was an effective agent that improved axonal degeneration in the patients [20]. In another study accomplished by Ward R et.al, DHA had a significant neuroprotective role in the axonal damage due to spinal cord injury [21]. With respect to DHA, it was conjugated with paclitaxel to form DHA-paclitaxel, a taxane-fatty acid conjugate with intratumoral activation. DHA-paclitaxel was more efficient treating cancer and caused significantly less toxicity over existing paclitaxel [22]. In addition, neurotoxicity may be prevented by cox-2 inhibition [23] and omega-3 fatty acids inhibit the generation of cox-2 mRNA [24].

US Food and Drug Administration (FDA) has recommended that a total maximum dose of 3 grams per day of DHA and EPA omega-3 fatty acids can be safely consumed [25]. In this study, the total dose of omega-3 fatty acids administered was 1244.1 mg per day (640 mg: 54% DHA, 10% EPA, three times a day) which was far below the maximum daily allowance of omega-3 fatty acids consumption for more caution. Although in a number of placebo-controlled prospective trials no considerable adverse effects were reported of omega-3 fatty acids, to prevent patients from experiencing nausea and gastrointestinal disturbances, they were advised to take fish oil pearls with meals and to keep them in a freezer [25]. There was a dramatic increase of serum concentrations of EPA & DHA in the group taking omega-3 fatty acid supplements and a significant difference was observed between two the study groups in terms of this issue, which could be an indicator of participant compliance.

## Conclusions

Although the survival rate of breast cancer patients improved by significant advances in treatment strategies, disabling and dose-limiting peripheral neuropathy due to chemotherapy with paclitaxel decreases the patients’ quality of life and sometimes forces the oncologist to change or even end the treatment [18]. No standard therapeutic agent exists for the prevention or treatment of chemotherapy-induced neuropathy [19]. Thus, finding a novel neuroprotective agent seems to be critical. Omega-3 fatty acids were efficient for prophylaxis against PIPN in this study. According to our knowledge, this is the first time that the efficacy of omega-3 fatty acids has been assessed for their ability to reduce incidence and severity of PIPN.

There were some limitations in the design of this double-blind placebo-controlled randomized trial. The lack of long term follow up of outcomes measured was a potential limitation of the study. In addition, Sun flower soft gelatin capsules used as placebo had no fishy taste, and the psychological status of the patients was not evaluated in this study although it may has been affected by omega-3 fatty acids. Relatively small sample size was another possible limitation of the current trial.

Polymorphisms of corresponding cytochrome P-450 enzymes could influence the paclitaxel clearance and drug-related side effects [17]. Therefore, genotyping could help to identify those cancer patients who are at risk of developing neurotoxicity so that they may be advised to take neuroprotectant supplements before the onset of painful and disabling PIPN. The effectiveness of omega-3 fatty acids supplementation for neuroprotection of patients with breast cancer treated with paclitaxel is supported by results from the present study. Another double-blind placebo-controlled randomized clinical trial with larger sample size is needed to confirm these findings.

## Abbreviations

PIPN, Paclitaxel-induced peripheral neuropathy; PN, Peripheral neuropathy; EPA, Eicosapentaenoic acid; DHA, Docosahexaenoic acid; PUFAs, Polyunsaturated fatty acids; rTNS, Reduced total neuropathy score; DML, Distal motor latency; a-CMAP, Compound muscle action potential amplitude; a-SAP, Sensory action potential amplitude; NCI-CTC, National cancer institute-common toxicity criteria; ECOG, European cooperative oncology group; QST, Quantitative sensory testing.

## Competing interests

The authors declare that they have no competing interests.

## Authors’ contributions

Zohreh Ghoreishi participated in the study design and coordinations and helped to prepare the draft of the manuscript and interpreting of the results, Ali Esfahani was the oncologist contributor of the study and helped to recruitment of the eligible patients and implementation the chemotherapy and intervention for the study subjects, Abolghasem Djazayeri participated in the study design, Mahmoud Djalali participated in the laboratory testing, Banafsheh Golestan participated in the study design from the statistical view of point, Hormoz Ayromlou performed the clinical and electrophysiological examinations, Shahriar Hashemzade and Vahid Montazeri participated in the recruitment of the eligible patients, Mahammad Asghari Jafarabadi, helped to analyze data and preparing tables and figures. Seyed ali Keshavarz participated in the study design, interpreting the results and he also helped to draft the manuscript. Masoud Darabi helped to set up the measuring the serum concentrations of omega-3 fatty acids.

## Pre-publication history

The pre-publication history for this paper can be accessed here:

http://www.biomedcentral.com/1471-2407/12/355/prepub
